# In silico vaccine design: Targeting highly epitopic regions of *Clostridium perfringens* type D epsilon toxin and *Clostridium novyi* type B alpha toxin for optimal immunogenicity^☆^

**DOI:** 10.1016/j.csbj.2024.08.009

**Published:** 2024-08-14

**Authors:** Nastaran Ashoori, Mohammad Mehdi Ranjbar, Romana Schirhagl

**Affiliations:** a Groningen University, University Medical Centre Groningen, Antonius Deusinglaan 1, 9713AW Groningen, the Netherlands; b Razi Vaccine and Serum Research Institute, Agricultural Research, Education and Extension Organization (AREEO), Karaj, Iran

**Keywords:** Vaccines, Machine learning, In silico, Toxins

## Abstract

Livestock infections caused by highly toxic bacteria, such as *Clostridium perfringens* type D and *Clostridium novyi* type B, present significant challenges in veterinary medicine. Such infections often require complex and elusive treatment regimens. Developing effective vaccines tailored to combat these specific pathogens remains a pressing need within the field. These bacteria are notorious for their extreme toxicity and the difficulty in culturing them for vaccine production. To address this challenge, we engineered a new potential vaccine candidate capable of neutralizing the virulence of both bacterial strains. Leveraging computational techniques, we identified epitopic regions within *C. perfringens* Epsilon Toxin (ETX) and *C. novyi* Alpha Toxin (ATX). Through fusion gene design, we integrated these epitopic regions alongside the PADRE-peptide sequence. The PADRE-peptide serves as a universal adjuvant to induce an immune response. The culmination of our efforts materialized in a Recombinant Fusion Protein D (rFPD), a novel vaccine construct designed to elicit robust and specific immune defenses against both bacterial species. By combining in-silico design and molecular engineering, our study represents a promising stride toward combating the impact of these pathogenic bacteria in livestock.

## Graphical Abstract

**Figure:**
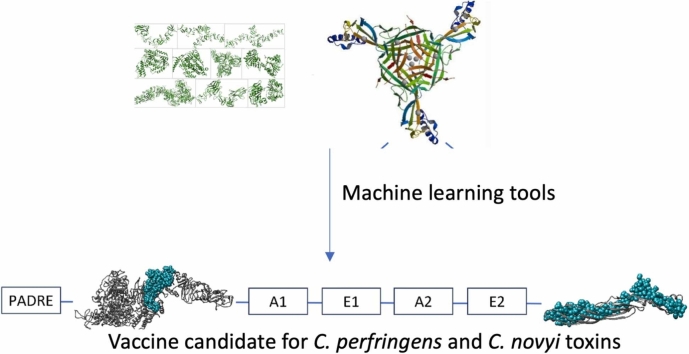

## 1 Introduction

Clostridia are a diverse group of gram-positive, anaerobic bacteria that form spores and lack motility, encompassing several pathogenic species. *Clostridium perfringens* is particularly notable for causing enteric diseases in domestic animals, leading to gastrointestinal disorders such as enteritis and enterotoxemia in species including cattle, sheep, goats, and pigs [48]. *C. perfringens* strains are classified into five types (A to E) based on the toxins they produce, with recent research identifying two additional types (F and G) [44].

*C. perfringens* type D is the primary causative agent of enterotoxemia in domestic ruminants. It produces a 33 kDa protoxin (328 amino acid residues). This protoxin undergoes cleavage and conversion into the active Epsilon toxin (ETX) by various proteases in the host’s gastrointestinal tract, including trypsin, chymotrypsin, and λ-protease encoded by *C. perfringens* [47]. The pore-forming ETX is considered the most toxic and fatal clostridial toxin after Botulinum and tetanus toxins. It plays a crucial role in the virulence of *C. perfringens* infections in sheep and goats [12], [15].

Enterotoxemia in lambs, sheep, and other livestock is commonly caused by consuming a highly concentrated, carbohydrate-rich diet, such as one high in cereals or lush pastures. These dietary changes disrupt the gut's microbial balance, leading to the presence of undigested fermentable carbohydrates, like starch, in the small intestine. This promotes the rapid proliferation of *C. perfringens*. The undigested starch causes glucose deficiency, which triggers the overproduction of Epsilon toxin (ETX) [42], [14]. Additionally, conditions that lead to intestinal stagnation can exacerbate the accumulation of *C. perfringens* and ETX in the intestinal loops. ETX can enter the systemic circulation and, during the enterotoxemic phase, bind to the microvascular endothelium in various organs. These include the brain, eyes, lungs, heart, liver, and kidneys, leading to toxic shock and potentially death [30], [40], [42], [53].

*Clostridium novyi* is the causative agent of several diseases in domestic animals like cattle, including bighead, black disease, bacillary hemoglobinuria, and red water [56], [33]. Based on the production of different toxins *C. novyi* is categorized into four types (A to D) [38], [39], [56]. Types A, B, and D are known to be pathogenic, while type C is generally considered non-pathogenic [32], [50], [49]. Specifically, *C. novyi* type B is responsible for Infectious Necrotic Hepatitis (INH), commonly referred to as black disease. This condition manifests as a peracute and fatal toxemic disease in domestic animals such as sheep, horses, pigs, and cattle [37], [5].

Spores of *C. novyi* type B, commonly present in soil and the gut microbiome of clinically healthy animals, can be spread through their feces. These spores are highly resilient to adverse environmental conditions. When ingested by grazing animals, the spores pass through the gastrointestinal tract and enter the bloodstream, eventually reaching vital organs such as the liver, spleen, and bone marrow. Within these organs, the spores are engulfed by local macrophages, where they can persist for extended periods. Liver damage creates anaerobic conditions that are conducive to spore germination and toxin production [5], [33].

The main virulence factor in *C. novyi* type B is Alpha toxin (ATX) [20], [33]. Following spore germination and the development of the vegetative state in the liver, ATX production leads to necrotic infarction and extensive hyperemia [13], [23], [24], [46], [48]. ATX is a 200–250 kDa protein with 2178 amino acid residues, belonging to the large clostridial cytotoxin family [21]. It acts as a glycosyltransferase on Rho proteins in target cells, disrupting the cytoskeleton by inhibiting signaling pathways, which results in increased vascular permeability and cell death [17], [2], [21], [7].

Infections caused by ETX and ATX progress rapidly. They often result in the death of affected animals shortly after exposure. Consequently, antibiotic treatment is ineffective because the swift progression of the diseases leaves little time for antibiotics to act. By the time clinical signs appear, the toxins have often already caused irreversible damage. Therefore, vaccination of domestic ruminants emerges as the most effective preventive measure [13]. Currently, immunization strategies rely on formaldehyde-inactivated toxins or toxoid-based vaccines. However, these vaccines often fail to elicit strong immunity against *C. perfringens* and *C. novyi*. Toxoid vaccines typically require large amounts or multiple doses to achieve adequate immunogenicity, and they frequently cause local reactions at the injection site [6]. Additionally, both bacteria are anaerobes, which complicates their cultivation and the preparation of toxins, making these processes labor-intensive and costly ([11], [17], [4], 2019b; [40]).

Recombinant DNA technology is a technique that involves using enzymes and different laboratory techniques to manipulate and isolate DNA segments. This approach allows the combination of DNA from different species or to creation of genes with new functions. In recent years, recombinant DNA technology has been increasingly employed to develop new generations of vaccines [55]. Using this approach, recombinant Epsilon Toxin (rETX) has been produced and evaluated as a potential vaccine candidate [9]. However, studies by Bokori-Brown et al. reported that rETX can cause local inflammation, highlighting the importance of precise dosage determination to prevent adverse effects. To mitigate these risks, it has been suggested to develop a smaller, highly immunogenic fragment of the toxin, which could provide a safer and more effective vaccine option [24].

Bioinformatics tools, software designed to extract meaningful information from biological data, have been employed to design epitope-based vaccines. These vaccines show promise compared to conventional vaccines due to their enhanced safety, stability, and ease of production. Additionally, the accurate identification of epitopes facilitates specific immune responses against pathogens, thereby reducing the risk of autoimmune responses and allergies [19], [28], [34], [35], [36], [52]. Recently, in-silico approaches— methods using computational approaches—have been increasingly utilized to accurately identify epitopes. These methods provide faster outputs compared to traditional experimental techniques and eliminate the risks and costs associated with culturing highly toxic bacteria [24], [34].

The rational genetic combination of genes encoding different proteins facilitates the production of polyvalent fusion proteins, such as multi-epitopic vaccines. Designing multi-target and highly efficient vaccines offers several advantages, including the incorporation of immunogenic B-cell and T-cell epitopes conserved across various pathogen variants into a single molecule. This approach enables the removal of non-protective but immune-dominant epitopes, the inclusion of adjuvants to boost immunogenicity, and the use of linkers to connect different segments and enhances the antigen presentation process [34], [36].

In the present study, we designed a fusion protein from the epitopic regions of ETX and ATX, targeting the simultaneous prevention of *C. perfringens* and *C. novyi* infections. We employed a rational design strategy, utilizing immunoinformatics and structural bioinformatics approaches to select and combine highly immunogenic regions. Specifically, we designed a novel fusion gene, *rfpd*, which encodes the rFPD protein. This protein includes highly immunogenic B-cell epitopes from both *C. perfringens* ETX and *C. novyi* ATX. To enhance the immunogenicity, we incorporated T-helper epitopes into the construct. Additionally, the PADRE-peptide was included as an adjuvant to further boost the efficacy and potency of rFPD as a potential vaccine candidate [51].

## 2 Materials and methods

**Fig. 1:**
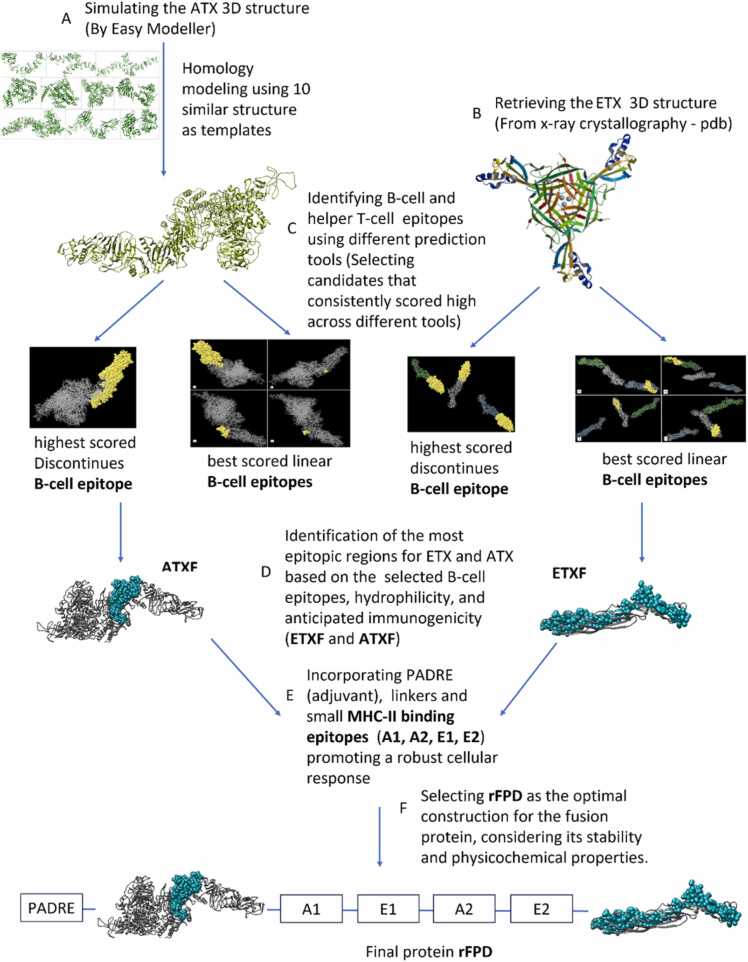
Illustrates the in-silico vaccine design process against *Clostridium perfringens* ETX and *Clostridium novyi* ATX. The steps are as follows: A: Predicting and simulating the 3D structure of ATX using EasyModeller 4.0. B: Retrieving the 3D structure (PDB) of ETX from the Protein Data Bank (these two steps are independent of each other and do not necessarily have to be done in this order). C: Identifying different B-cell and helper T-cell epitopes of ETX and ATX using various prediction tools (Immune Epitope Database (IEDB), TepiTool, CBS, Prediction server, LBTope, SVMTriP, CBS-Bepipred, ABCpred, CBTope, DiscoTope). D: Selecting the most immunogenic regions of ETX and ATX, based on B-cell epitopes identified in step C, hydrophilicity, and predicted immunogenicity, using CLC Main Workbench 5.5. E: Enhancing the construct by adding PADRE (adjuvant), linkers, and small MHC-II binding epitopes (derived from step C), to stimulate a robust cellular response, designated as A1, A2, E1, E2. F: Selecting the rFPD construct for the fusion protein based on its stability and physicochemical properties, analyzed using EXPASY-ProtParam.

In this study, we employed immunoinformatic analysis to design a potential vaccine candidate against *C. perfringens* ETX and *C. novyi* ATX using in-silico methods. A schematic overview of the vaccine design process is illustrated in Fig. 1.

### 2.1 Determination of 3D structures for conformational B-cell epitope prediction

To predict the conformational B-cell epitopes of ETX and ATX, we used the 3D structures of these toxins. The complete 3D model of *C. perfringens* ETX was retrieved from the Protein Data Bank (PDB) under PDB ID: 1UYJ (https://www.rcsb.org/structure/1UYJ). For *C. novyi* ATX, whose 3D structure is not available from crystallographic studies or related reports, we employed EasyModeller 4.0 software [26] to generate a model based on homology modeling and multiple threading alignments. This software constructs 3D structures using the structural data of homologous proteins found in existing databases.

To identify suitable toxin-like PDB structures for ATX modeling, we performed a BLASTP search against the PDB database using the ATX amino acid sequence (NCBI-BLASTP 2.6.1, https://blast.ncbi.nlm.nih.gov/Blast.cgi). From the results, we selected 10 proteins with amino acid sequence similarities exceeding 30 % compared to ATX. Given this similarity, we deemed homology modeling appropriate for generating a 3D model of ATX. The Swiss-Pdb Viewer 4.1.0 software (https://spdbv.unil.ch/) was used to perform energy minimization of the predicted model. Subsequently, we assessed the model’s accuracy through Ramachandran plot analysis using the RAMPAGE server [27]. The validated model was then used for predicting conformational B-cell epitopes.

### 2.2 Primary analysis of ETX and ATX sequences

The nucleotide and amino acid sequences for both toxins were retrieved from the NCBI database (https://www.ncbi.nlm.nih.gov/) and analyzed using NCBI BLAST. For *C. perfringens* ETX, we used the reference sequence YP_002291114.1 and accession number KU726256.1. For *C. novyi* ATX, the reference sequence Z48636.1 and accession number CAA88565.1 were employed. We obtained and assessed the secondary structure of both toxins, including β-sheet and α-helix formations, as well as their hydrophobicity (using the Kyte-Doolittle algorithm) and antigenicity (predicted with the Kolaskar-Tongaonkar algorithm) graphs using CLC Main Workbench 5.5 (https://clc-genomics-workbench.software.informer.com/5.5/). Areas of the proteins that are exposed and hydrophilic were identified as having high potential immunogenicity and were considered as B-cell epitopes.

### 2.3 Identification of the MHC-II binding epitopes

To predict helper T-cell binding MHC-II epitopes, we utilized several bioinformatics tools, including the Immune Epitope Database (IEDB) MHC-II Binding Predictions server (http://tools.iedb.org/mhcii/), TepiTool (http://tools.iedb.org/tepitool/), and the CBS Prediction server (NetMHCII 2.2) [8]. We selected the H2-IAd allele, the most prevalent allele in BALB/C mice [45], given that BALB/C mice were chosen as the animal model for subsequent experimental analysis. Among these tools, IEDB was identified as the most comprehensive tool for epitopic prediction algorithms [29], making its results particularly reliable. Nonetheless, to ensure the accuracy of the selected epitopes and validate IEDB’s predictions, we cross-verified the outcomes with those from other servers.

### 2.4 Linear B-cell epitope prediction

For the prediction of linear epitopes of B-cell lymphocytes, the protein sequences of both toxins were submitted to various servers, including LBTope (http://crdd.osdd.net/raghava/lbtope/), SVMTriP (http://sysbio.unl.edu/SVMTriP/index.php), CBS-Bepipred (https://services.healthtech.dtu.dk/services/BepiPred-2.0/), ABCpred (http://crdd.osdd.net/raghava/abcpred/), IEDB-Bepipred, IEDB-Kolaskar, and IEDB-Emini surface accessibility (http://tools.iedb.org/bcell/). These servers utilize a combination of physicochemical properties of amino acids and machine learning algorithms to identify potential linear B-cell epitopes.

### 2.5 Conformational B-cell epitope identification

The PDB file (3D structure) of ETX (PDB ID: 1UYJ) and the final models of ATX were analyzed using several servers to predict conformational B-cell epitopes. The servers utilized include CBTope (http://crdd.osdd.net/raghava/cbtope/), DiscoTope-2.0-CBS (https://services.healthtech.dtu.dk/services/DiscoTope-2.0/), and Ellipro-IEDB (http://tools.iedb.org/ellipro/). CBTope employs the Support Vector Machine (SVM) approach to calculate amino acid composition and identify residues involved in antibody interactions [3]. DiscoTope-2.0-CBS utilizes surface accessibility and residue propensity scores in the spatial context of the protein [25]. Ellipro-IEDB predicts discontinuous epitopes by assessing residue accessibility and flexibility, providing a Protrusion Index (PI) score [54].

Following B-cell epitope predictions, we selected one highly antigenic fragment from each toxin that exhibited overlap in both continuous and discontinuous predicted epitopes. These fragments were identified as the most immunogenic regions of the toxins. To assess their properties, antigenicity and hydrophobicity as well as the secondary structures of the selected ETX and ATX fragments were obtained using CLC Main Workbench 5.5. These were then compared with the corresponding properties of the full-length toxins to determine whether the fragments maintain or mimic the functional and structural characteristics of the whole toxin.

### 2.6 The design of the recombinant fusion protein

The final selected B-cell epitopic fragments, along with the two most immunogenic helper T-cell epitopes (MHC-II Binding Epitopes) for each toxin, were utilized to construct the fusion protein. Appropriate linkers were chosen to fuse these segments. These linkers served as spacers to facilitate the correct folding of conformational epitopes within the chimeric proteins. The selection of linkers was based on their proven effectiveness in promoting the proper folding and stability of multi-epitope proteins in previous studies. Specifically, the NH₂-GPGPG-COOH linker was used to connect the MHC-II binding epitopes, while the NH₂-GGSSGG-COOH linker was employed for the B-cell epitopic fragments. These linkers were selected for their flexibility, ability to prevent steric hindrance, and demonstrated utility in similar constructs, as reported by Negahdaripour et al. [34], Nezafat et al. [36], and Ranjbar et al. [43].

The organization of the helper T-cell epitopes in the fusion protein was determined based on their scores obtained from the IEDB server, which is recognized as a comprehensive resource for epitope prediction algorithms [29]. This approach was based on the principle that positioning a stronger epitope adjacent to a weaker one can enhance the overall immunodominance. To further increase the immunogenicity of the recombinant fusion protein, the PADRE sequence (universal T-helper Pan DR Epitope: AKFVAAWTLKAAA) was incorporated at the N-terminus. This sequence is known for its ability to bind to various MHC-II alleles strongly and non-specifically. This approach reduces the risk of vaccine failure and functions as an adjuvant [16], [2].

Five different models of the recombinant fusion protein, namely Recombinant Fusion Protein A-D (rFPA, rFPB, rFPC, rFPD, and rFPE), were evaluated for their ability to fuse epitopes from both toxins. The assessment focused on their immunogenicity and physicochemical properties, including molecular weight, pH, half-life, solubility, and stability. These properties were analyzed using the EXPASY-ProtParam tool (https://web.expasy.org/protparam/). Additionally, potential protein allergenicity was investigated using AlgPred (http://crdd.osdd.net/raghava/algpred/) and AllergenFP v.1.0 (https://ddg-pharmfac.net/AllergenFP/) databases, given that the selected model is intended to be non-allergenic. The final selection of the best model, rFPD, was based on its favorable structural features and a comprehensive evaluation of all physicochemical properties.

### 2.7 Reverse translation and sequence verification of rFPD

The rFPD protein sequence was reverse-translated into a nucleotide sequence. For this process, the nucleotide sequences corresponding to the selected MHC-II binding epitopes and B-cell epitopic regions were derived from the NCBI sequences for ETX (Accession Number: KU726256.1) and ATX (Accession Number: Z48636.1). However, the linkers and the PADRE peptide were reverse-translated using the Back-Translation tool from the EMBOSS database (https://www.ebi.ac.uk/jdispatcher/st/emboss_backtranseq). Subsequently, the JCat server [18] was used for optimizing codon usage, CG content, and restriction enzyme avoidance. The start codon (ATG) and stop codon (TAG) were incorporated into the constructs (*rfpd* gene) to ensure proper translation initiation and termination.

To assess the presence of restriction enzyme sites within the *rfpd* gene, the final nucleotide sequence was analyzed using CLC Main Workbench 5.5. This step is essential because identifying restriction sites helps in selecting the most suitable enzymes for cloning the gene into the vector. Additionally, to ensure the accuracy of the final sequence, it was translated into a protein sequence using CLC Main Workbench 5.5. The resulting protein sequence was then compared with the designed protein using BLAST on the MultAlin server (http://multalin.toulouse.inra.fr/multalin/).

## 3 Results

### 3.1 Primary analysis of the ETX and ATX sequences

The DNA and protein sequences of ETX and ATX, as analyzed through NCBI-Blast (https://www.ncbi.nlm.nih.gov/), demonstrate a high degree of conservation. This suggests that vaccine candidates incorporating epitopes from both toxins could be effective against various strains of *C. perfringens* type D and *C. novyi* type B. For ETX, the hydrophobicity plot generated by CLC Main Workbench 5.5 (https://clc-genomics-workbench.software.informer.com/5.5/) indicates that, excluding the signal peptide (residues 1–32), the region around residues 150–300 is the most hydrophilic and likely to be highly immunogenic (It is marked with circles in Supplementary Material Appendix 1, Fig. S1). Additionally, secondary structure analysis shows that residues beyond 135 aa predominantly form loops and β-sheets (Supplementary Material Appendix 1, Fig. S2). Consequently, the region between residues 150–300 is identified as the most promising epitopic area.

For ATX, the hydrophobicity plot generated by CLC Main Workbench 5.5 shows that the most hydrophilic region spans approximately between residues 1100 to 1700 (Supplementary Material Appendix 1, Fig. S3-A). Although the antigenicity plot suggests various potential antigenic regions (Supplementary Material Appendix 1, Fig. S3-B), we prioritized the epitopes within this more hydrophilic section. Secondary structure prediction for ATX indicates that residues 1069–2178 are predominantly composed of β-sheets and loops (Supplementary Material Appendix 1, Fig. S4). This region is considered promising for containing highly antigenic epitopes, as loops and β-sheets are generally more antigenic than α-helices [22].

### 3.2 Identification of MHC-II binding epitopes

MHC-II binding epitopes were predicted using the IEDB server (http://tools.iedb.org/mhcii/), TepiTool (http://tools.iedb.org/tepitool/), and the CBS Prediction server [8] for the prevalent MHC allele in mice (H2-IAd). These servers provide numerical scores for the epitopes, with lower scores indicating stronger binding affinity. The results from these various servers were compared for both ETX and ATX. Based on the IEDB results, which are considered the most reliable, the two most immunogenic epitopes for each toxin were selected (Supplementary Material Appendix 1, Table S1).

### 3.3 Linear B-cell epitope prediction

To predict linear B-cell epitopes of ETX and ATX, various servers were utilized, including LBTope (http://crdd.osdd.net/raghava/lbtope/), SVMTriP (http://sysbio.unl.edu/SVMTriP/index.php), CBS-Bepipred (https://services.healthtech.dtu.dk/services/BepiPred-2.0/), ABCpred (http://crdd.osdd.net/raghava/abcpred/), IEDB-Bepipred, IEDB-Kolaskar, and IEDB-Emini surface accessibility (http://tools.iedb.org/bcell/). The predictions were performed with a threshold value set at 0.6, which helped identify significant epitopic regions. For ETX, residues 150–300 were predicted to contain prominent linear B-cell epitopes, while for ATX, the C-terminal region spanning residues 1200–2178 was identified as encompassing more continuous B-cell epitopes (see Supplementary Material Appendix 1, Table S2 and Table S3).

The global antigenicity value for each protein segment was calculated by summing the contribution values of individual residues within the predicted epitopic regions. These contribution values are based on various parameters such as hydrophilicity, flexibility, accessibility, turns, exposed surface, polarity, and antigenic propensity, which are assessed using propensity scales for each of the 20 amino acids. The score for each residue is calculated by averaging the scale values of neighboring residues within a specified window size, typically 5 to 7 residues long, centered on the residue of interest. This method allows the identification of regions with higher potential antigenicity, guiding the selection of epitopes for further validation (http://tools.iedb.org/bcell/help/).

### 3.4 Determination of 3D structures for conformational B-cell epitope prediction

**Fig. 2:**
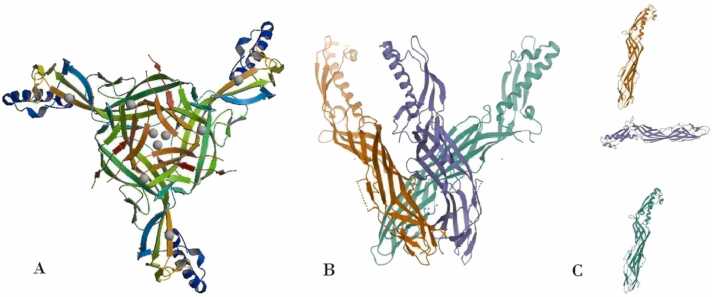
Shows the 3D structure of *C. perfringens* Epsilon Toxin (ETX). The structure was retrieved from the Protein Data Bank with the PDB ID: 1UYJ (ETX-PDB). The structure provides insights into the spatial arrangement of ETX molecules. A and B represent the Biological Assemblies, depicting how the ETX chains interact and form larger structures. C corresponds to the Asymmetric Unit, illustrating the smallest repeating unit of the crystal structure.

To predict discontinuous B-cell epitopes, servers require the 3D structure of the antigenic protein. The Protein Data Bank (https://www.rcsb.org/structure/1UYJ) provided the 3D structure for ETX (ETX-PDB), which is available under PDB ID: 1UYJ [12] (Fig. 2).

**Fig. 3:**
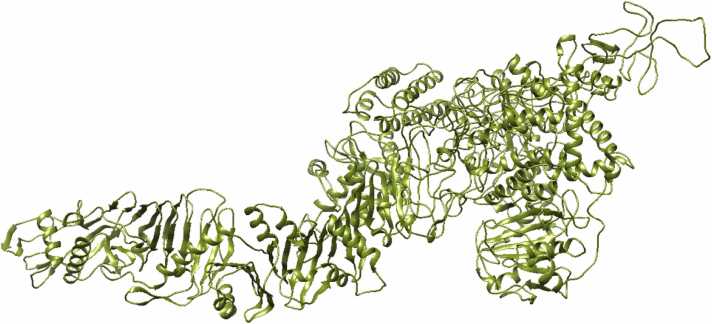
Represents the *C. novyi* ATX 3D model predicted by EasyModeller 4.0 software using homology modeling (ATX-3D).

**Fig. 4:**
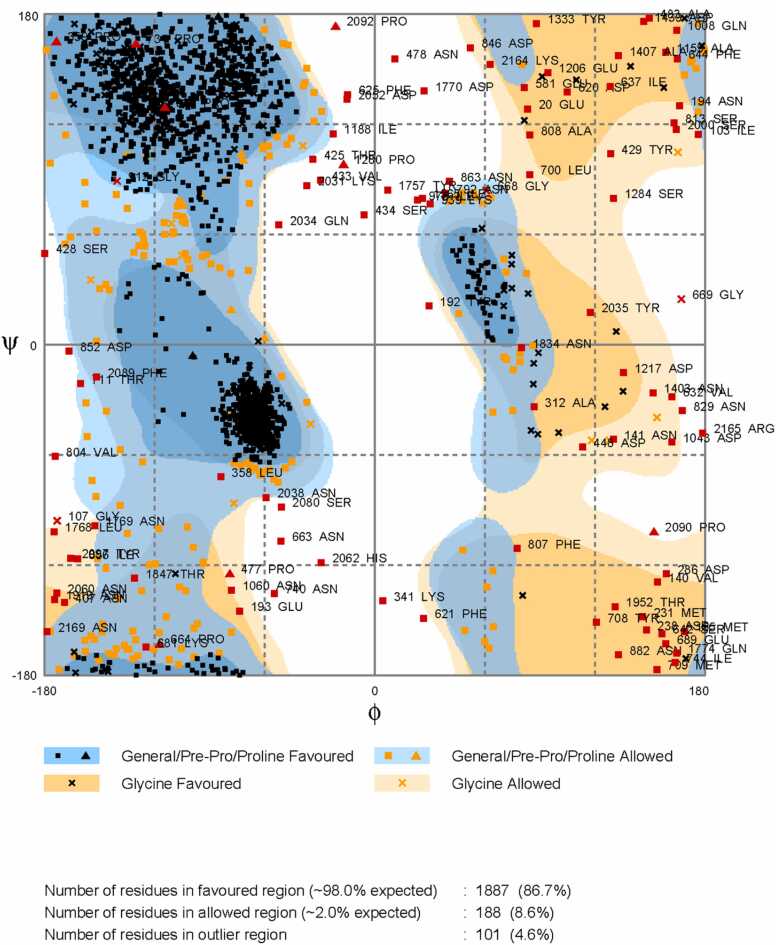
Displays the Ramachandran plot for ATX-3D generated by RAMPAGE. This diagram illustrates the ϕ versus ψ backbone conformational angles in proteins. It showcases observed conformations or calculated energy contours for dipeptides. In recent years, these plots have become vital for structure validation, serving as a sensitive indicator of local issues since ϕ, ψ values are not optimized in the refinement process. The Ramachandran diagram, summarizing information on backbone conformation, has significantly contributed to our comprehension of protein structure, energetics, and folding. The plot indicates that 1887 residues (86.7 %) fall within the favored region, 188 residues (8.6 %) in the allowed region, and 101 residues (4.6 %) in the outlier region. Considering that 95.3 % of residues lie in the valid area, the ATX-3D model can be deemed ideal [27].

For ATX, which lacks a crystallography-derived 3D model, EasyModeller 4.0 [26] was used to generate a 3D model (ATX-3D) through homology modeling (Fig. 3). Following energy minimization with Swiss-Pdb Viewer 4.1.0 (https://spdbv.unil.ch/), the structure validation of ATX-3D was assessed using a Ramachandran plot from the RAMPAGE server [27] (Fig. 4). The plot indicates that 1887 residues (86.7 %) are in the favored region, 188 residues (8.6 %) are in the allowed region, and 101 residues (4.6 %) are in the outlier region. With 95.3 % of residues within valid regions, the ATX-3D model is considered to be of high quality.

### 3.5 Conformational B-cell epitope identification

**Fig. 5:**
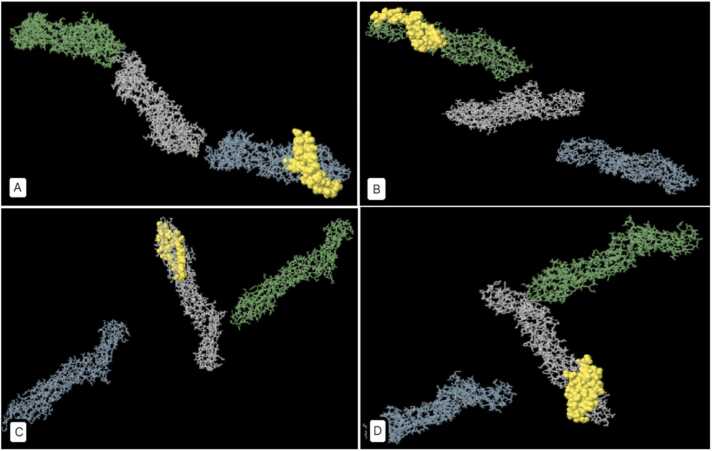
Depicts a 3D representation of the highest-scoring Linear B-cell epitopes of *C. perfringens* ETX, as predicted by IEDB-Ellipro (highlighted in yellow). A: Chain A's highest-scoring Linear B-cell epitope spans residues 246–295 (PI score: 0.786). B: Chain B's highest-scoring Linear B-cell epitope encompasses residues 195–239 (PI score: 0.719). C and D: Chain C boasts two top-scoring Linear B-cell epitopes (C: Residues 159–184, PI score: 0.882 - D: Residues 250–295, PI score: 0.875). Additional details can be found in Supplementary material Appendix 1, Table S2.

**Fig. 6:**
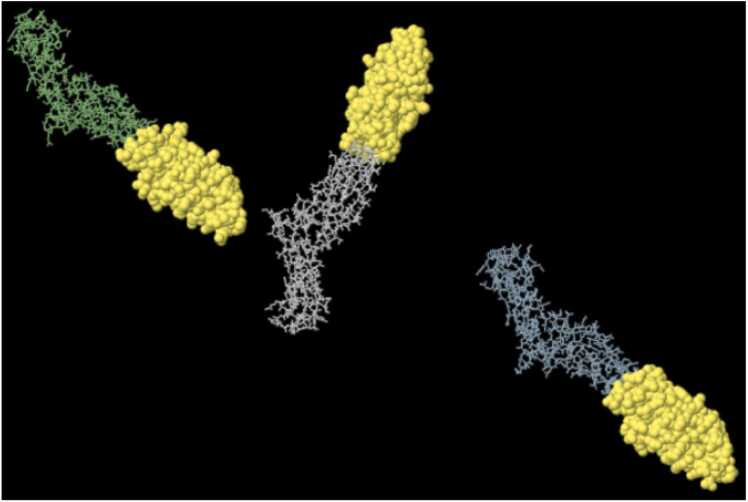
Shows the 3D representation of the highest scored (PI score: 0.856) Discontinuous B-cell epitope of *C. perfringens* ETX by IEDB - Ellipro (Details in Supplementary material Appendix 1, Table S4).

**Fig. 7:**
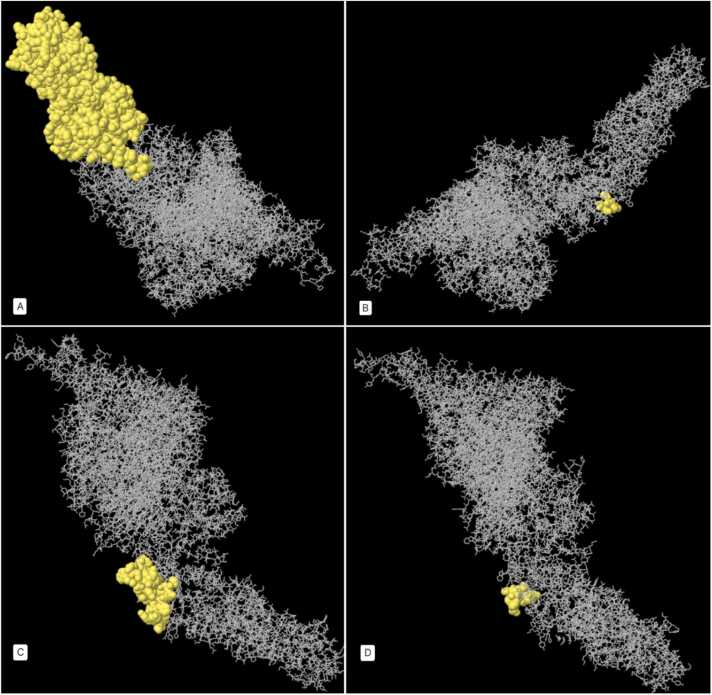
Is a 3D illustration of the high-scored Linear B-cell epitopes of *C. novyi* ATX by IEDB-Ellipro. A: The top-scoring Linear B-cell epitope spans residues 1375–1697 (PI score: 0.839). B: A high-scoring Linear B-cell epitope encompasses residues 1846–1852 (PI score: 0.717). C: Another high-scoring Linear B-cell epitope is found in residues 1862–1907 (PI score: 0.695). D: A high-scoring Linear B-cell epitope is present in residues 1829–1839 (PI score: 0.688). (More details in Supplementary material Appendix 1, Table S4).

**Fig. 8:**
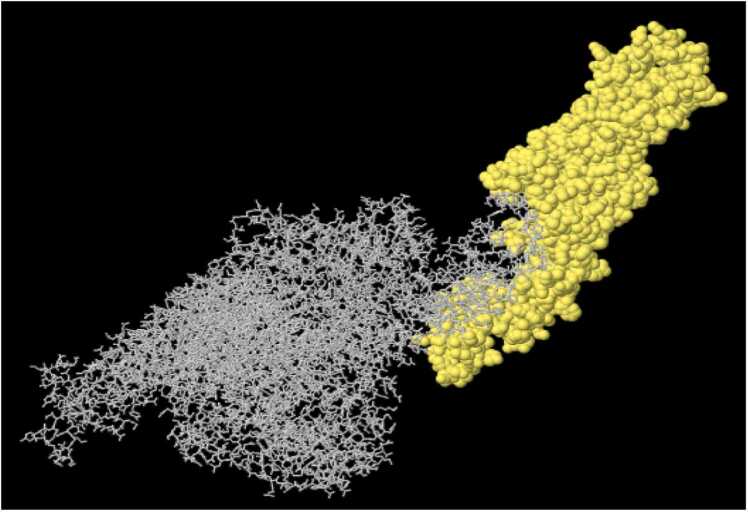
Depicts the highest score (472aa, PI score: 0.779) Discontinuous B-cell epitope of *C. novyi* ATX by IEDB - Ellipro in yellow (Details in Supplementary material Appendix 1, Table S5).

Conformational B-cell epitopes for ETX-PDB (excluding residues 1–32 as the signal peptide) and ATX-3D (all 2178 residues) were predicted using three databases: CBTope (http://crdd.osdd.net/raghava/cbtope/) with a threshold of −0.3, DiscoTope-2.0-CBS (https://services.healthtech.dtu.dk/services/DiscoTope-2.0/) with thresholds of −0.37 and 0.5, and Ellipro-IEDB (http://tools.iedb.org/ellipro/) with a threshold of 0.5. The results are summarized in Table S4 and Table S5 (Supplementary material Appendix 1). ElliPro, the prediction tool, utilizes ellipsoids to approximate the 3D structure of the protein antigen. Each epitope is associated with a Protrusion Index (PI) score, signifying the proportion of protein residues within a specific ellipsoid. Higher PI values imply a larger inclusion of residues in the epitope, indicating enhanced solvent accessibility and potential antigenicity. Ellipro outputs were visually represented as 3D structures in Fig. 5, Fig. 6, Fig. 7, Fig. 8.

**Fig. 9:**
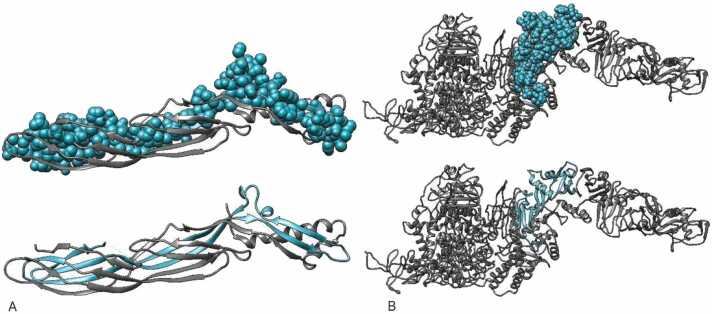
Presents a 3D representation of the selected epitopic fragments of A: *C. perfringens* ETX (ETXF) and B: *C. novyi* ATX (ATXF) using UCSF Chimera 1.8. The ribbon and surface visualization provides a comprehensive depiction of the spatial arrangement and structural characteristics of the chosen epitopes. The ribbon representation highlights the backbone structure of the epitopes, offering insights into their overall conformation, while the surface visualization provides a detailed view of the molecular surface features. UCSF Chimera 1.8, a versatile molecular visualization tool, facilitates the exploration and analysis of these epitopic fragments in three dimensions, aiding in a more thorough understanding of their structural attributes.

Based on predictions for continuous and discontinuous B-cell epitopes, as well as antigenicity and hydrophobicity plots and secondary structure analyses of the toxins, the most immunogenic regions were identified. For ETX, the most epitopic region spans residues 200–300, while for ATX, it covers residues 1800–2178. The highly immunogenic fragments selected were ETXF (residues 199–302) for ETX and ATXF (residues 1822–1992) for ATX. The 3D molecular structures of ETXF and ATXF were visualized using ribbon and surface representations in the UCSF Chimera 1.8 visualization system for further exploratory analysis [41] (Fig. 9).

### 3.6 Design of the recombinant fusion protein

**Fig. 10:**
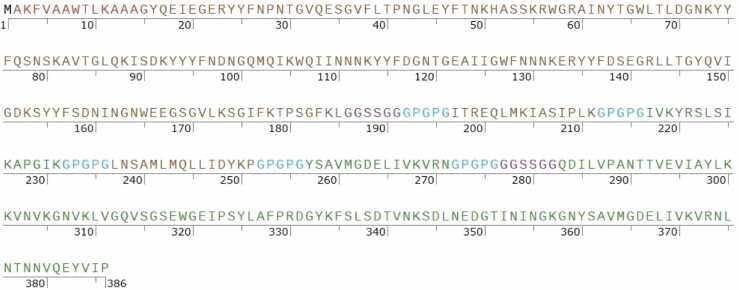
Illustrates the amino acid sequence of the selected Recombinant Fusion Protein D (rFPD) derived from epitopic fragments and MHC-II binding epitopes of *C. perfringens* ETX and *C. novyi* ATX. The sequence consists of 386 amino acid residues and starts with methionine attached to the N-terminal end as initial amino acid. The adjuvant, PADRE sequence, is highlighted in red for clarity. TEF, representing T-helper Epitopic Fragment, encompasses regions A1, E1, A2, and E2, crucial for eliciting immune response. ATXF, denoting selected fragments from *C. novyi* ATX, is depicted in brown, along with A1 and A2, the highest-scoring Helper T-cell epitopes for ATX. Similarly, selected fragments from *C. perfringens* ETX, ETFX, and E1 and E2, the top-scoring Helper T-cell epitopes for ETX, are depicted in green. GGSSGG serves as linker for B-cell epitopes, shown in purple, while the linker for Helper T-cell epitopes contains the GPGPG sequence, represented in blue.

To construct a recombinant fusion protein (rFP) incorporating ETXF, ATXF, E1, E2, A1, A2, and the PADRE peptide as an adjuvant, we evaluated five different models (A-E). The T-helper Epitopic Fragments (TEFs) were created by combining the MHC-II binding epitopes (A1, E1, A2, E2), with the linker GPGPG to connect each T-helper epitope. These epitopes were arranged in descending order of their immunogenic scores to optimize the immune response. To ensure proper folding of the B-cell epitopic fragments within the recombinant protein, the GGSSGG linker was employed. This linker not only promotes correct folding but also serves as a site for targeted proteolytic cleavage in the bloodstream. Models A-E were evaluated as potential candidates for the fusion protein (see Fig. 10 and Supplementary material Appendix 1, Table S6).

The physicochemical properties of Recombinant Fusion Protein D (rFPD) were evaluated using EXPASY-ProtParam (https://web.expasy.org/protparam/). rFPD has a molecular weight of 42 kDa and an isoelectric pH of 9.09. It demonstrates a suitable in vitro half-life of 30 h and an in vivo half-life of over 10 h in *E. coli*. The instability index of rFPD is 20.71, indicating it is a stable protein (with an instability index below 40). The GRAVY (Grand Average of Hydropathicity) index for rFPD is −0.443, suggesting that it is immunogenic. While all tested constructs exhibited similar physicochemical properties, rFPD was selected as the most promising vaccine candidate because the ATX and ETX domains are spatially distant from each other, preventing interference with their respective structures. Details of the rFPD protein sequence are provided in Table S8 (Supplementary material Appendix 1). Additionally, analyses using AlgPred (http://crdd.osdd.net/raghava/algpred/) and AllergenFP v.1.0 (https://ddg-pharmfac.net/AllergenFP/) confirm that rFPD is non-allergenic.

### 3.7 Reverse translation of the recombinant fusion protein D (rFPD)

The reverse translation process for Recombinant Fusion Protein D (rFPD) involved several critical steps to ensure the accurate reconstruction of the nucleotide sequence. Initially, nucleotide sequences for the ETXF, ATXF, and TEF fragments were derived from their respective toxins. The sequences for the linkers and the PADRE peptide were reverse-translated using BackTranseq-EMBOSS (https://www.ebi.ac.uk/jdispatcher/st/emboss_backtranseq). To adapt codon usage, optimize GC content, and analyze restriction enzyme sites, the JCat server [18] was utilized. A start codon (ATG) and a stop codon (TAG) were incorporated into the sequence, resulting in a total nucleotide length of 1161 bp for the *rfpd* gene (Supplementary material Appendix 1, Table S9). Following this, the *rfpd* sequence was translated into a protein using CLC Main Workbench 5.5. Verification of the reverse translation was performed by comparing the reconstructed protein sequence with the designed rFPD protein using the MultAlin server (http://multalin.toulouse.inra.fr/multalin/).

**Fig. 11:**
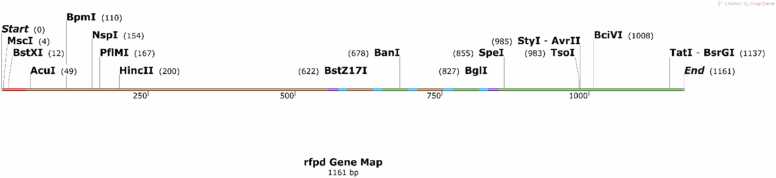
Presents the reverse translation of the Recombinant Fusion Protein D (rFPD) into the corresponding nucleotide sequence, referred to as the *rfpd* gene. The nucleotide sequence of the *rfpd* gene comprises 1161 base pairs (bp). The initiation codon "ATG" signifies the start codon located at the N-terminal end, initiating the translation process, while the termination codon "TAG'' denotes the stop codon positioned at the C-terminal end, signifying the end of protein synthesis. Highlighted in red, the adjuvant PADRE sequence is prominently displayed for clarity, indicating its pivotal role in enhancing immunogenicity. Additionally, the table delineates specific regions within the *rfpd* gene, such as the T-helper Epitopic Fragment (TEF), comprising regions A1, E1, A2, and E2, crucial for eliciting an immune response. Moreover, the table categorizes selected fragments from *C. novyi* ATX, as ATXF, depicted in brown, along with the highest-scoring Helper T-cell epitopes (A1 and A2) for ATX. Similarly, ETFX, selected fragments from *C. perfringens* ETX, denoted as ETFX, are represented in green, along with the top-scoring Helper T-cell epitopes (E1 and E2) for ETX. The nucleotide sequence of the linkers for B-cell epitopes is depicted in purple, facilitating the structural integrity and functionality of the fusion protein. The sequence for the linkers of Helper T-cell epitopes is represented in blue, ensuring proper spatial arrangement and interaction with immune cells. The *rfpd* gene contains several restriction enzyme sites, which impact the selection of appropriate restriction enzymes for future cloning. To ensure precise cutting and avoid unwanted cleavage, it is essential to choose restriction enzymes that do not cut within the *rfpd* gene itself.

The analysis from CLC Main Workbench 5.5 revealed several restriction enzyme recognition sequences within the *rfpd* gene, which is crucial for molecular cloning. This identification is important because it informs us which restriction enzymes to avoid during the cloning process. Using restriction enzymes that recognize these sequences could lead to unintended cleavage and potential disruption of the gene sequence. Consequently, this insight assists in selecting alternative restriction enzymes that will not interfere with the integrity or functionality of the *rfpd* gene construct (Fig. 11).

## 4 Discussion

*Clostridium perfringens* type D causes fatal enteric diseases, while *Clostridium novyi* type B is responsible for lethal gangrene in domestic ruminants. The principal virulence factors in these diseases are the *C. perfringens* type D Epsilon toxin (ETX) and the *C. novyi* type B Alpha toxin (ATX) [20]. Given the high fatality rate associated with these bacteria and the limited time available for antibiotic intervention, vaccination emerges as the most viable alternative for preventing these infections.

Conventional vaccines, particularly toxoids, face challenges such as undesirable side effects, non-specific immune responses, and the complex, costly manufacturing process associated with anaerobic bacteria. To overcome these issues, modern immune informatics techniques are utilized to identify novel immunogenic regions and epitopes within target proteins. This innovative approach aims to generate specific and targeted immune responses, facilitating cost-effective production methods for various bacterial pathogens.

In this study, various immunoinformatic tools were employed to design a novel Recombinant Fusion Protein (rFPD) serving as a vaccine candidate against both *C. perfringens* type D and *C. novyi* type B simultaneously. The rFPD incorporates optimal epitopic fragments from ETX (ETXF: residues 199–302) and ATX (ATXF: residues 1822–1992), along with the two most immunogenic T-helper epitopes from each toxin (E1, E2, A1, A2). These epitopes were selected based on T-helper and B-cell epitope databases, secondary structure analysis, and hydrophobicity and antigenicity plots. Additionally, the PADRE peptide (AKFVAAWTLKAAA) was included as an adjuvant to enhance rFPD's immunogenicity. Strategic linkers were implemented to connect the fragments, avoiding the formation of new epitopes, preventing interference among selected epitopes, and mitigating potential issues related to immunodominance.

The selection of antigenic determinants is pivotal for designing vaccines that elicit effective immune responses. Previous research has demonstrated that coil structures on a molecule's surface and β-sheets are more likely to act as antigenic determinants, generating stronger immune responses compared to α-helix structures [16], [31]. In line with these findings, the chosen epitopic fragments ETXF and ATXF, derived from loop-like and β-sheet regions identified through immunoinformatics databases, were selected to enhance the vaccine's antigenicity. Additionally, selecting fragments based on overlapping continuous and conformational B-cell epitope regions further supports their potential as highly antigenic components.

The confirmation of our bioinformatics analysis through the inclusion of highly antigenic continuous B-cell epitopes is supported by recent research by Alves et al. [1]. Their study identified four highly antigenic continuous B-cell epitopes among 15 potential candidates for developing subunit vaccines against *C. perfringens* type D ETX. Notably, ETXF, the antigenic fragment selected for ETX in our design, contains three of these confirmed epitopic determinants. This region is crucial due to its role in toxin attachment to the membrane and its involvement in the interaction among the three ETX chains to form the cell pore. Antibodies targeting this region could potentially prevent ETX activation by inhibiting the cleavage of the protoxin by enzymes.

The alignment of our selected fragment, ATXF (1822–1992 aa), with the highly antigenic C-terminal segment of ATX, specifically pNTF12 (1800–1958 aa), reinforces the validity of our immunoinformatic design. Research by Busch et al. [10] and Gord-Noshahri et al. [17] identified pNTF12 as a hydrophilic surface protein crucial for receptor binding. This segment is noted for its high mobility and absence of catalytic activity. Importantly, our chosen ATXF fragment significantly overlaps with this critical pNTF12 region, supporting its potential as an effective antigenic target.

Gord-Noshahri et al. [17] demonstrated that anti-NTF12 antibodies actively recognized pNTF12 in immunological assays. These antibodies, produced in mice, showed not only a strong recognition of ATX but also higher reactivity towards native ATX compared to anti-ATX antibodies. This finding suggests that the 3D structure and epitopes of pNTF12—and by extension, ATXF—are likely preserved in their native conformation.

## 5 Conclusions

In conclusion, our in-silico design of Recombinant Fusion Protein D (rFPD) represents a promising vaccine candidate capable of offering simultaneous immunity against both *Clostridium perfringens* type D and *Clostridium novyi* type B. This fusion peptide integrates the optimal epitopic fragments from Epsilon toxin (ETX) and Alpha toxin (ATX), along with two highly immunogenic T-helper epitopes from each toxin (E1, E2, A1, A2). The inclusion of the PADRE peptide as an adjuvant is expected to further enhance the immunogenicity of rFPD.

The subsequent critical steps involve synthesizing and evaluating rFPD through animal trials to assess its effectiveness and protective efficacy. These experiments will provide essential insights into the potential of rFPD as a viable vaccine candidate for preventing infections caused by *C. perfringens* type D and *C. novyi* type B.

## Author Contributions

N. Ashoori conducted all the experiments in this article and wrote the first draft under supervisions of M.M. Ranjbar and R. Schirhagl. All authors have read and agreed on the final version of the paper.

## CRediT authorship contribution statement

**Romana Schirhagl:** Writing – review & editing, Supervision. **Mohammad Mehdi Ranjbar:** Supervision. **Nastaran Ashoori:** Writing – original draft, Formal analysis, Data curation, Conceptualization.

## Declaration of Competing Interest

RS is founder of QTsense. The activities of QTsense are unrelated to this manuscript. The other authors have no conflict of interest to declare.
